# Summary of the proceedings of the international forum 2016: “Imaging referral guidelines and clinical decision support - how can radiologists implement imaging referral guidelines in clinical routine?”

**DOI:** 10.1007/s13244-016-0523-4

**Published:** 2017-01-02

**Authors:** European Society of Radiology (ESR)

**Affiliations:** European Society of Radiology (ESR), Neutorgasse 9/2, 1010, Vienna, Austria

**Keywords:** Imaging referral guidelines, Clinical decision support, ACR Appropriateness Criteria, ESR iGuide

## Abstract

**Abstract:**

The International Forum is held once a year by the ESR and its international radiological partner societies with the aim to address and discuss selected subjects of global relevance in radiology. In 2016, the issue of implementing imaging referral guidelines in clinical routine was analysed. The legal environment in the USA requires that after January 1, 2017, physicians must consult government-approved, evidence-based appropriate-use criteria through a clinical decision support system when ordering advanced diagnostic imaging exams. The ESR and the National Decision Support Company are developing “ESR iGuide“, a clinical decision support system for European imaging referral guidelines using ESR imaging referral guidelines based on ACR Appropriateness Criteria. In many regions of the world, the situation is different and quite diverse, depending on the specific features of health care systems in different countries, but there are, unlike in the USA and EU, no legal obligations to implement imaging referral guidelines into the clinical practice. Imaging referral guidelines and clinical decision support implementation is a complex issue everywhere and the legal environment surrounding it even more so; how they will be implemented into the clinical practice in different areas of the world needs yet to be decided.

***Main messages*:**

• *Implementation of imaging referral guidelines in clinical routine is needed*.

• *Potential benefits are improved appropriateness in referrals and reduction of unnecessary radiation exposure*.

• *The educational benefits include new insights through data collection and reporting*.

• *The system will potentially highlight the lack in quality or availability of equipment*.

## Introduction

The International Forum (originally Summit) was established by the European Society of Radiology (ESR) in order to intensify the collaboration of national and international radiological societies from outside Europe and to discuss selected subjects of global relevance in radiology at each European Congress of Radiology (ECR). At the 2013–2015, the relation between radiology and nuclear medicine, the position of ultrasound in radiology, and the relation of general radiology and subspecialty radiology were discussed. At the ECR 2016, the implementation of clinical decision support (CDS) and imaging referral guidelines in the clinical routine was discussed.

Representatives of the following radiological societies, usually the president and one executive member, were invited to this meeting to present the situation in their respective country or region: The American College of Radiology (ACR), the Asian Oceanian Society of Radiology (AOSR), the Canadian Association of Radiology (CAR), the Colombian Association of Radiology (ACR), the Indian Radiological and Imaging Association (IRIA), the Inter-American College of Radiology (CIR), the International Society of Radiology (ISR), the Japan Radiological Society (JRS), the Korean Society of Radiology (KSR), the Radiological Society of North America (RSNA), the Royal Australian and New Zealand College of Radiologists (RANZCR), and the European Society of Radiology (ESR). Representatives of several past “ESR meets” countries/societies were also invited to attend the meeting.

The use of high-tech diagnostic imaging procedures, like magnetic resonance imaging (MRI), computed tomography (CT), positron emission tomography (PET), and nuclear cardiology tests, has seen double-digit increases annually in the last decade. While remarkable technological advances have enabled precise imaging of many complex physiologies and often ensure more accurate diagnostics, concerns are being raised that the sharp increase in these tests does not necessarily correspond proportionately to improved patient outcomes.

The ACR Appropriateness Criteria®, in existence for more than 20 years, define guidelines for the most appropriate medical imaging exam for a patient’s condition, if any imaging is needed at all. These comprehensive criteria cover 215 topics and more than 1080 clinical indications. Appropriateness criteria use has been shown to improve quality, reduce unnecessary imaging and lower costs. They are widely accepted because they are created and continually updated by panels of physician experts from many different medical specialties. Implementation of a CDS system (CDSS) in the USA is needed because of the legal obligations, but the interest is considerable in other parts of the world.

The development of European guidelines started at the end of 2014, with the scientific review of ACR Select™ content by ESR experts and the adaptation of ACR appropriateness criteria to European standards of practice was performed. The process in Europe is developing quite fast. In the other parts of the world, the situation is diverse, but radiologists generally recognize advantages of imaging referral guidelines and CDS in clinical practice, but are also aware of potential drawbacks.

### The Situation in North America

The American College of Radiology (ACR) perspective was presented by Bib Allen.

In the year 1993, the ACR was already prepared to define a system of patient care guidelines for radiology to eliminate inappropriate utilization of radiologic services and to produce substantial savings to the health-care system, without a negative impact on the quality of care. It was indicated radiologists need to take leadership roles in defining the most beneficial services for patients and those that are most cost-effective [1].

The ACR Appropriateness Criteria® required more than 20 years of work to develop, included the input of hundreds of multi-specialty-based clinical experts and almost 6000 literature references that are constantly updated, fully transparent and widely referenced. Appropriateness criteria use has been shown to improve quality, reduce unnecessary imaging and lower costs. They are widely accepted because they are continually updated and the evidence is based on a full spectrum of multi-specialty scientific literature — not solely radiology. Providers of all specialties can easily and confidently use ACR Appropriateness Criteria® to help improve the quality of care that they provide. It was shown that the provider acceptance of prior authorization programs is poor, because the criteria are not transparent; the only education is to learn to game the system, which results in delayed diagnosis and disrupts the physician–patient relationship, and it causes administrative costs. To deliver CDS to providers, evidence-based criteria are required as well as standardised methodology based on diverse sources of information that are continuously updated and transparent to providers, with suggestions for alternative examinations, a robust set of clinical indications and the digital capture of the care experience. Health information technology is a key to the future of public health surveillance, patient safety, healthcare quality measurement and delivery system improvement. CDS needs to be integrated into the clinical workflow, electronic health records (EHR) and order entry system. An EHR-embedded software solution or standalone software platform is needed. Maintenance and technical support are crucial. The content updates need to be distributed seamlessly, and site customizations need to be locally modifiable. End-user feedback is very important.

Bib Allen demonstrated examples from the Medicare Imaging Demonstration Project and implementation in Massachusetts General Hospital. The Centers for Medicare & Medicaid Services have named the ACR a “qualified Provider-Led Entity” approved to provide appropriate use criteria (AUC) under the Medicare Appropriate Use Criteria program for advanced diagnostic imaging. This means that medical providers can consult ACR Appropriateness Criteria® to fulfill impending the Protecting Access to Medicare Act (PAMA) requirements in that they consult AUC prior to ordering advanced diagnostic imaging for Medicare patients. According to the PAMA, after January 1, 2017, physicians must consult government-approved, evidence-based appropriate-use criteria through a CDS system (CDSS) when ordering advanced diagnostic imaging exams (CT, MRI, NM and PET). The U.S. Department of Health and Human Services (HHS) through CMS is authorized to deem specific appropriate-use criteria for CDS. The criteria must be scientifically valid, evidence-based, and based on studies that are published and reviewable by the stakeholder.

ACR Select is now integrated into the EHR in 90 % of the providers’ systems. The order is opened within an EHR, wherin the ordering physician records a structured reason for the exam using ACR Select, and conducts a search for and selection of indications. ACR Select presents guidance and scores, and generates a decision support number and records the appropriateness of the order within the EHR. The appropriate exams require no additional workflow steps. In the National Radiology Data Registry medical imaging results, recommendations and outcomes are collected, which is important for education, provides metrics for value-based care, and enables determination of outliers.

The timeline for CDS implementation in the USA is settled. Bib Allen concluded that the CDS encompasses a robust and localizable set of guidelines covering all modalities and the vast majority of indications, that the electronic order entry solution needs to be embedded in EHR and that the registry is important for referrer education and accountability.

Richard Baron of the RSNA reemphasized that the PAMA is a law passed in the USA in 2014 that requires ordering providers to consult a computerized CDSS that covers outpatient and emergency CT, MRI, and nuclear medicine studies. No reimbursement will be possible without documentation of consultation, and the details of implementation were left to the Centers for Medicare & Medicaid Services (CMS).

Several aspects of CDS implementation were discussed, for example, how are the AUC created and certified, what is the minimum set that must be consulted, what are the minimum required software mechanism features and how are they certified, and how will the compliance be documented and verified. Regarding the AUC creation, the established criteria for entities need to be certified. All AUC created by certified entities are considered certified by the CMS, and applications are accepted annually on January 1, with 20–30 entities having applied for this first year. The issue of the minimum required coverage of AUC was presented. Two major options exist: broad coverage, and staged implementation starting with a small set of AUC for which there is strong evidence. The first option has the potential to reduce inappropriate imaging more, but was unsuccessful in the Medicare Imaging Demonstration Project, and likely requires expert opinion-based AUC. The latter option may have better referring provider acceptance. Regarding the software mechanisms, at least three systems are commercially available. There is a diversity of opinions on minimum features illustrated by a range of feature sets in these systems. This is needed to have a simple match of indication and imaging type, leading directly to an appropriateness score, and the branching logic is needed to determine appropriateness based on clinical data from electronic medical record (EMR) and provider. None of the software was certified at the time of the meeting, as the CMS has not yet developed requirements or processes.

Richard Baron concluded that CDS implementation is complex and the policy surrounding it even more so, that many different entities with differing perspectives are involved in AUC creation and software development, and that most of the important issues that will set the requirements for how CDS is implemented in the USA are yet to be decided.

William Miller, representative of the CAR presented the situation in Canada, the current state, and what is on the horizon. Studies performed in Canada have shown quite variable data, so that the rate of inappropriate studies ranges from 1.5 % to 24 %.

The CAR Referral Guidelines were revised in 2013, and considerable work and international cooperation was performed to keep them evidence-based and current [2]. The guidelines are available online and in print and are presented to various general practitioners (GPs) and referring physicians at CMEs across Canada. But, they are rarely used. Dr. Miller emphasized that the CDS should be part of the Computerized Physician Order Entry (CPOE) systems, so when a referring physician orders a test, instant feedback regarding the appropriateness is provided and the alternatives are suggested if a test demonstrates low appropriateness. An evidence base should be available at a click, and all should be integrated within the routine workflow.

Dr. Miller emphasized that the European iGuide, which is based on the ACR Appropriateness Criteria in ACRSelect, is revised in light of European practice, and may be a closer fit for Canada. Regarding the penetration of the CDS in the CPOE systems, currently, the penetration of electronic medical records in Canada is in the range of 60–70 %, and CDS in CPOE is now possible in most Canadian regions. The expected benefits for Canada are the improved appropriateness of examinations, reduction in unnecessary radiation exposure, streamlined clinical workflow, and shortened wait lists. Educational benefits are important due to the feedback on the appropriateness of selected exams and new insights through data collection and reporting. The system can also highlight the lack of availability of imaging.

In January 2016, the CAR Board approved exploration of collaboration with the ESR and the ACR to develop a unique Canadian product based on ACR Select/iGuide, with the goal to have the product in one year. The new Canadian government is willing to spend money to stimulate the economy, is willing to spend on innovation, and is interested in improving healthcare in Canada. The CAR believes that CDS in CPOE is innovative and is working for support.

### The situation in Latin America

A survey about how radiologists can implement imaging referral guidelines into the clinical routine was completed by 10 Latin American member societies of the Interamerican College of Radiology/Colegio Interamericano de Radiología (CIR), and CIR representative Dante R. Casale Menier presented the analysis of the answers obtained by three groups of examinees: radiologists, medical school students and other medical specialists (members of non-radiologic speciality societies). Radiologists emphasized the need to have adequate access to ACR guidelines, European guidelines and local (country) guidelines. They recognize the need to adapt the guidelines according to the technology available at each location where they work. This is needed to develop and implement quality programs in every radiology department and integrate guidelines into radiology information systems. Radiologists would like to be able to review through the system the most relevant topics from radiology meetings and courses and to have a relevant bibliography available. The medical students emphasized the value of imaging referral guidelines for education, on the undergraduate level as well, and the standardization of teaching. They believe that imaging referral guidelines (IRG) could strengthen case-based clinical practice by reporting on a real case. Other medical specialists think that such guidelines should be distributed to clinical doctors, that emergency services would benefit considerably, and are willing to have access to ACR and European guidelines on their web pages.

Federico G. Lubinus of the ACR presented the point of view of his association. The arguments in favour of introducing IRG are that they provide the best available evidence, reduce the error rate, and protect against lawsuits. The arguments against IRG are that they may be potentially biased by economic interests, that they constrain medical acts, and may be, to a certain point, outdated. In Colombia, the so called “Methodological Guide for the development of Clinical Practice Guidelines with Economic Evaluation for the Colombian public Health System“ exists, authored by four physicians and five non-physicians. The work to create this guide took 10 years, and some guidelines could be controversial because some of the technologies included are not covered by the insurance benefits. They also probably became outdated during the 10 years of creation.

Dr. Lubinus cited the president of the Colombian Association of Scientific Societies, who said that the evidence-based medicine (EBM) is used to make "explicit recommendations for specific situations, based on statistically validated research", and that it became the basis for developing practice guidelines, which was misused by auditors and administrative staff and even by uninformed doctors, who converted the EBM in an instrument to constrain the medical act. The Colombian Statutory Law of Health from 2015 establishes medical autonomy and self-regulation as the main principles of the law. The autonomy of health professionals is guaranteed to make decisions about the diagnosis and treatment of patients they are responsible for. This autonomy shall be exercised within the framework of self-regulation schemes, ethics, rationality and scientific evidence. All constraints, pressure or restriction of practice that threatens the autonomy of the health professionals, as well as any abusive practice that threatens patient safety is prohibited.

The ACR point of view is that medical autonomy requires self-regulation, and for this, one needs to fit in a professional practice based on the best available evidence standards, which should be freely adopted by professionals, without constraint, but, in formal adhesion. The ACR thinks that radiologists can implement imaging referral guidelines in clinical practice by taking away any economical bias, the guidelines should be reviewed permanently by peers, and self-regulation and formal adhesion should be achieved by strengthening education.

### The situation in Australia and New Zealand

Greg Slater, representative of RANZCR, presented the situation in Australia and New Zealand. The RANZCR currently does not have an official set of imaging guidelines or decision support tools. However, there is an increasing need for an official set with approaches from internal, domestic sources, as well as international sources. The independent, high-level task force was established to provide strategic advice in developing a college position on imaging guidelines and decision support tools. It surveyed key stakeholders to identify the need for a set of guidelines, the need to evaluate existing resources and the need to assess what role RANZCR should play.

The recommendations of the Task Force are the following: (1) the RANZCR Board acknowledges the need for and potential benefits of bi-national imaging referral guidelines for Australia and New Zealand and reiterates the college’s commitment to leading efforts to promote best practice in imaging referrals; (2) RANZCR commits to leading a multidisciplinary effort to improve the availability and use of imaging referral guidelines by referrers and imaging providers in Australia and New Zealand; (3) the RANZCR Board does not consider the de novo development of guidelines; (4) the RANZCR Board investigates the option to endorse and/or adopt and adapt an existing guideline tool for bi-national implementation; (5) the RANZCR Board develops a strategy for RANZCR to take a leadership role in engaging with other stakeholders, particularly imaging referrers, to support implementation of imaging referral guidelines and decision support tools. The RANZCR Board endorsed all five recommendations of the Task Force.

RANZCR is committed to reducing inappropriate imaging and ensuring all imaging is appropriate and clinically accountable. A list of six imaging procedures that clinicians and consumers should question was developed for the Choosing Wisely Australia initiative in 2015. The six recommendations will be submitted to the Choosing Wisely New Zealand initiative in 2016. Two recommendations are co-branded with the Australasian College for Emergency Medicine (ACEM). Choosing Wisely Australia is an initiative of NPS (National Prescribing Service) MedicineWise. Choosing Wisely New Zealand is an initiative of the Council of Medical Colleges (CMC). The six recommendations are the following: (1) Don’t request imaging for acute ankle trauma unless indicated by the Ottawa Ankle Rules (localised bone tenderness or inability to weight-bear as defined in the rules); (2) Don’t request duplex compression ultrasound for suspected lower limb deep venous thrombosis in ambulatory outpatients unless the Wells score (deep venous thrombosis risk assessment score) is greater than 2, OR if less than 2, and the D dimer assay is positive; (3) Don’t request any diagnostic testing for suspected pulmonary embolism (PE) unless indicated by Wells score (or Charlotte rule) followed by PE rule-out criteria (in patients not pregnant). Low-risk patients in whom diagnostic testing is indicated should have PE excluded by a negative D dimer, not imaging; (4) Don’t perform imaging for patients with non-specific acute low back pain and no indicators of a serious cause for low back pain; (5) Don’t request imaging of the cervical spine in trauma patients, unless indicated by a validated clinical decision rule (joint recommendation with the ACEM); (6) Don’t request computed tomography head scans in patients with a head injury, unless indicated by a validated clinical decision rule (joint recommendation with the ACEM).

Supplementary resources were developed to support the Choosing Wisely recommendations: clinical decision rules, a clinical decision rules application (currently only for iOS) and a recommendations poster. Educational modules for appropriate imaging topics are: introduction to clinical decision rules (CDR), adult head trauma, paediatric head trauma, adult cervical spine trauma, paediatric cervical spine trauma, acute low back pain, suspected pulmonary embolism, suspected lower limb deep vein thrombosis, acute ankle trauma in adults, and paediatric ankle trauma. Implementation requires adequate advocacy in terms of delivering better healthcare, providing the best tests available at the right time, reviewing radiation exposure, and improving the cost/benefit ratio. There are some barriers, both internal and external, to the college. Internal barriers encompass resources (manpower, expertise, finances), maintenance, sustainability, review, viability, governance, research, membership involvement, deployment and audit, while external barriers refer to the involvement of key stakeholders, access, governmental and regulatory bodies, liability and patient consent.

### The situation in India

O.P. Bansal presented the situation in India and the point of view of the IRIA.

Dr. Bansal pointed out that the duty of radiologists is to provide safe patient care services which improve the outcome of the patient. The IRIA is in line with other organizations with regard to imaging referral guidelines for radiologists, and believe they are needed because of the overuse and inappropriate use of imaging, increasing costs and radiation exposure risks. The IRIA also recognizes that some of its members may find aspects of imaging referral guidelines challenging in the current scenario. The guidelines should assist them to address the challenges, and to achieve the common goal of ensuring that primary care has proper access to high-quality imaging services as a negative investigation can exclude disease and put the patient at risk. Criteria for developing guidelines should take into account modality and age, issues of optimizing radiation dose and cost-effectiveness. Imaging technique guidelines are needed for different modalities, and also for different clinical situations, as well as for reporting.

The referral pattern in India is such that the treating physician refers the case to the radiologist with a request to carry out a specific diagnostic investigation. Generally, the case is not discussed with the radiologist prior to sending the patient to decide the appropriate modality of investigation. The radiologist generally carries out the investigation desired by the physician and writes a report; recommendations regarding further investigations are also given, if required. The IRIA has nominated academics of repute as heads of various subspecialties, who were asked to prepare a white paper regarding imaging guidelines for various common clinical scenarios prevalent in Indian settings. The academic wing of the IRIA is engaged in formulating imaging guidelines for a wide variety of conditions and there is a regular interaction between the referring clinician and the radiologist by inviting them to various conferences and CME organized by the IRIA to make them aware of the recent advances in radiology and help them to choose the appropriate investigation in a given clinical setting.

### The situation in Japan and Korea

H. Honda of the JRS pointed out that the Japanese health care system is unique regarding CT and MRI systems, because Japan has the highest number of CT and MR machines per population in the world, double the amount of the USA. Also, almost all costs of CT and MRI studies, including contrast are covered by the governmental universal health insurance. The Health, Labour and Welfare Ministry of Japan supports the development of imaging referral guidelines and the creation of a clinical decision support system incorporating these guidelines. It also supports the trial to assess implementation of the CDSS in hospitals. The JRS has its own imaging guidelines, and plans to incorporate into their guidelines the existing referral guidelines (e.g., ACR appropriateness criteria), that will be tailored to the Japanese situation (availability of imaging equipment, disease prevalence, Japanese standards of practice, etc.). The translation into the Japanese language has already been performed.

Regarding the creation of a CDSS, the software will be integrated into Japanese hospitals’ existing IT infrastructure. The SS-MiX2 (Standardized Structured Medical record Information exchange), the Japanese standardized data format, will be used to extract basic patient data (demographics, information on previous exams etc.) from the EHR and imaging referral guidelines will be incorporated [3]. As a conclusion, imaging referral guidelines and a clinical support system have just started in Japan, and the trial of CDSS in hospitals will be implemented within a couple of years.

The KSR representative, Seung Hyup Kim, presented the basic data of the Korean healthcare system that is in all aspects regulated by the government. The National Health Insurance system is a public insurance system with mandatory registration of all healthcare providers and citizens and provides universal coverage. The Health Insurance Review Agency (HIRA) reviews healthcare provider reimbursement claims and decides the reimbursement amount and coverage application. Reimbursement is based on the ‘fee-for-service’ system, and imaging is performed by prescription, not by referring, meaning that if the order is entered, it is almost always performed. Radiologists are involved in protocol management, but have little role in the decision-making about the examination. The fee per service is low compared with many OECD countries, resulting in increased volumes, or delivery of services outside the health insurance by providers. Korea is characterized by easy accessibility to all levels of hospitals with low fee per hospital visit, and it is easy to get imaging studies done.

Clinicians have concerns about the use of guidelines because of regulatory and reimbursement issues. There is also inertia among clinicians to change practice patterns according to guidelines, and the lack of knowledge about appropriate imaging for the given clinical circumstances. An important issue is also the practice of defensive medicine. Patients on the other side request fast diagnosis, require special studies, perform so called “hospital shopping“, and the demand for health screening is increasing.

From the radiologists’ standpoint, the active involvement in the decision-making about the exam is not a part of radiologist work in daily practice, and they often consider the involvement in decision-making as mere extra paperwork. Among radiologists in Korea, there is a lack of awareness of the presence of clinical imaging referral guidelines. Also, with the implementation of imaging guidelines, exam volumes would decline, leading to a decrease in income and tightening the job market for radiologists which can be a threat for the specialty of radiology. Developing imaging guidelines takes time and needs experienced personnel which can be a burden for radiologists.

Nevertheless, the KSR is trying to incorporate imaging guidelines into radiological practice in the country and has established an Imaging Guideline Committee . Education programs are performed about guideline development. The KSR plans to develop comprehensive clinical imaging guidelines in collaboration with the National Evidence-based Healthcare Agency (NECA) and Korea Centers for Disease Control. The development of a ‘Korean imaging referral guideline’ and ‘repeat imaging guideline’ can serve as baseline for HIRA review, and an audit will be performed of previously released guidelines to identify its utilization, reasons for not following guidelines, and actual change in practice behaviour. Currently available imaging referral guidelines in Korea are: guidelines for the use of cardiac CT in cardiac disease (2012), CT guidelines for repeat imaging (2013), Korean guidelines for appropriate utilization of cardiovascular MRI (2014). The ongoing guideline projects supported by the KSR are the development of comprehensive clinical imaging guidelines, an abdomen CT guideline for patients admitted to emergency rooms with acute abdomen issues, diagnosis and management of nodules found on lung cancer screening (clinical practice guideline), and recommendations for common paediatric fluoroscopy exams.

As a conclusion, the KSR has started to develop clinical imaging guidelines, and the development of a clinical decision support system has not been started yet, but will follow the development of guidelines. KSR will implement newly developed guidelines in diverse ways in cooperation with governmental regulatory bodies.

### The situation in Asia and Oceania

K. Prabhakar Reddy of the AOSR emphasized the need for imaging referral guidelines because of the overuse and inappropriate use of imaging, increasing costs and radiation exposure. In his opinion, the easy access to MRI/CT scanners may be linked to the high rate of unnecessary investigations. Two-thirds of the MRI scans appear to result from increased availability within the first month of the onset of back pain and clinical guide lines recommend delaying MRI use until four weeks after the onset, during which time most low-back pain patients show spontaneous improvement. Reasons for inappropriate imaging are many, and include patients’ expectations and demands for imaging, conflict of interest presented by physician ownership of imaging equipment, incentives for referrals, lack of specific guidance from radiologists and lack of knowledge by referring physicians and other providers and non-physicians. In his opinion, family practice physicians frequently order unnecessary tests and as many as 30 % of diagnostic imaging procedures are inappropriate or contribute no useful information.

Dr. Reddy emphasized that radiologists must act as consultants to the referring physicians, that the recognised criteria could help in the selection of proper examination, and that integration of imaging guidelines into the radiological information system could help for appropriate imaging requests. The AOSR, based on Dr. Reddy’s presentation, supports the development of referral guidelines and a CDSS to ensure appropriate imaging now and in the future.

### Situation in developing countries – position of the International Society of Radiology

J.P. Borgstede of the ISR reported that the mission of the ISR as a representative non-governmental organization (NGO) is to facilitate the global endeavours of the ISR’s member organizations to improve patient care and population health through medical imaging. In developing countries, the basic needs are to provide food, water, clothing, shelter and safety, and the medical care comes on top of these basic needs. In medical care, primary care delivery is most important; in many instances, reusable equipment is used for imaging, and the referral guidelines and the decision support system are at the end of the list of needs. Many developing countries have only radiography and perhaps ultrasound, and appropriateness of whether to image or not to image is important, but often determined by the availability of a certain modality.

Providers in developing countries do very good physical exams, and imaging interpretation is predominantly performed by individuals with limited training who may not even be physicians. So, the issues of infrastructure, equipment, personnel and safety are crucial. Therefore, the developing countries are not in the stage of adopting imaging referral guidelines and CDS, but Dr. Borgstede concluded that the key to the success of referral guidelines and CDS is that these are a local product that involves referring physicians who accept the product, that they must be electronically facile and commercially available in the EHR.

### The situation in Europe

The perspective of the ESR was presented by its president, Luis Donoso. The ESR-led European Commission study in the year 2013 demonstrated the inadequate status quo with limited availability of imaging referral guidelines in Europe, since 70 % of countries had some type of guidelines. Only the UK and France had fully developed guidelines, while the others adapted and adopted these guidelines. It was determined that imaging referral guidelines are hardly used in daily practice. On the other side, the EURATOM Basic Safety Standards Directive (passed in 2013 and applicable from February 2018) mandates guideline availability in all member states of the European Union.

The approach of the ESR to the issue was developed over the last years. The ESR deemed merging different national guidelines in Europe to be unfeasible and, consequently, established a partnership with the ACR to “Europeanise“ appropriateness criteria for use in Europe. Regarding the selection of the clinical decision support software, the National Decision Support Company (NDSC) emerged as the only viable platform provider. The ESR and NDSC are developing “ESR iGuide“, the clinical decision support system for European imaging referral guidelines using ESR imaging referral guidelines based on ACR appropriateness criteria, i.e. the ACR Select system and content [4].

The development of European guidelines started in November 2014, with the scientific review of ACR Select content by ESR experts, which was overseen by the dedicated methodologist. The adaptation of ACR appropriateness criteria to European standards of practice was performed. The generic European imaging referral guidelines provide a common core standard across Europe for further localisation on national or institutional bases. Periodic content updates of ESR iGuide and ACR Select will be performed in cooperation between ESR and ACR. The ESR Imaging Referral Guidelines Working Group under the ESR Quality, Safety and Standards Committee was established at ECR 2016 and the ACR Rapid Response Committee has consisted of joint membership of ACR and ESR experts since May 2016.

The basic transactional workflow consists of selecting the imaging test, entering the reason for the exam, and receiving feedback. For each access to the criteria, the system generates a unique decision support number. Several pilot tests are planned in 2016. The pilot integration process consists of five phases. Phase 0 is the preparatory phase and scope definition. Phase 1 consists of translation, system building and testing. Phase 2 consists of mapping organisational procedures to ESR iGuide content. Phase 3 consists of testing and changing management. Phase 4 consists of “going-live“ and the collection of feedback and usage statistics. The envisaged locations of pilot sites are: Belgium, Croatia, France, Germany, Ireland, Italy, Russia, Spain, Sweden, the Netherlands and the UK.

Regarding the strategy for implementation, it is important that the introduction of CDS for referral guidelines must be a hospital project driven by a radiology department, but sponsored by management and all stakeholders, especially referrers, must be involved early on. Adequate integration into existing software/workflows is essential for adoption, which requires close cooperation of IT departments and IT providers. Translation, review and, if necessary, localisation of referral guidelines is essential for their adoption. Depending on the specific regional situation, informing/involving political stakeholders (local/regional authorities, regulators, social insurance, health ministries, etc.) may be important.

So, to briefly summarize, the timeline in Europe was the following: in December 2013, the ESR-led European Commission study on referral guidelines was published. The ESR-ACR-NDSC partnership was announced at ECR 2014. In November 2014, the guideline europeanisation started, and the demo version was launched at ECR 2015. In the autumn of 2015, the European content was finalized and in October 2015, NDSC Europe was established in Vienna, Austria, and soon the joint ESR-ACR updating process started. At ECR 2016, the above-mentioned ESR Imaging Referral Guidelines Working Group was established, and pilot tests in European countries started in 2016.

The expected benefits are improved appropriateness in medical imaging referrals, reduction in unnecessary radiation exposure, a more reliable justification process, better accountability through a structured workflow, and a streamlined clinical workflow. Also, the educational benefits are obtained through the feedback on appropriateness of the selected exam and new insights through data collection and reporting. The system will help to demonstrate the utility of imaging equipment and will potentially highlight a lack in quality or availability.

## Discussion

The legal environment in the USA (Protecting Access to Medicare Act, PAMA) requires that after January 1, 2017, physicians must consult government-approved, evidence-based appropriate-use criteria through a CDSS when ordering advanced diagnostic imaging exams. The U.S. Department of Health and Human Services is authorized to deem appropriate specific appropriate-use criteria for clinical decision support. ACR Select is now integrated into the electronic health record in 90 % of providers’ systems in the USA. The ACR Appropriateness Criteria® required more than 20 years of work to develop, included hundreds of multi-specialty-based clinical experts and almost 6000 literature references, and they are constantly updated, fully transparent and widely referenced. Appropriateness criteria use has been shown to improve quality, reduce unnecessary imaging and lower costs.

In Europe, only the UK and France had fully developed guidelines, and the EURATOM Basic Safety Standards Directive (passed in 2013 and applicable from February 2018) mandates guideline availability in all member states of the European Union. The ESR and the NDSC are developing “ESR iGuide“, the clinical decision support system for European imaging referral guidelines using ESR imaging referral guidelines based on ACR appropriateness criteria, i.e. the ACR Select system and content.

In many regions of the world, the situation is different and quite diverse, depending on the specific features of health care systems in different countries, but there are, unlike in the USA and EU, no legal obligations to implement imaging referral guidelines into the clinical practice.

In Canada, it is considered that the European iGuide, based on the ACR appropriateness criteria in ACR Select, that is revised in light of European practice, may be a closer fit for Canada. In South America, the need for imaging referral guidelines is recognized, but they are not widely used. In Australia and New Zealand, the need for and potential benefits of imaging referral guidelines for Australia is recognized, and the RANZCR is committed to leading a multidisciplinary effort to improve the availability and use of imaging referral guidelines by referrers and imaging providers and will investigate the option to endorse and/or adopt and adapt an existing guideline tool for bi-national implementation. A list of six imaging procedures that clinicians and consumers should question was developed for the Choosing Wisely Australia initiative in 2015. In India, the radiological association has asked academics of repute as heads of various subspecialties to prepare a white paper regarding imaging guidelines for various common clinical scenarios prevalent in Indian settings. Japan has its own imaging guidelines, and plans to incorporate into their guidelines the existing referral guidelines, that will be tailored to the Japanese situation (availability of imaging equipment, disease prevalence, Japanese standards of practice, etc.). In Korea, the development of clinical imaging guidelines was started, while the development of a clinical decision support system has not been started yet, but will follow the development of guidelines. The newly developed guidelines will be implemented in cooperation with governmental regulatory bodies. The developing countries are not in the stage of adopting imaging referral guidelines and clinical decision support.

Imaging referral guidelines and clinical decision support implementation is a complex issue everywhere and the legal environment surrounding it even more so; how they will be implemented into clinical practice in different areas of the world needs yet to be decided.

## Conclusions

The need for the implementation of imaging referral guidelines in clinical routine is recognized among the radiology community worldwide, and the process is most advanced in the USA and the European Union.

The expected benefits are improved appropriateness in medical imaging referrals, reduction in unnecessary radiation exposure, a more reliable justification process, better accountability through a structured workflow, and a streamlined clinical workflow. The educational benefits will be obtained through the feedback on appropriateness of selected exams and new insights through data collection and reporting. The system will help to demonstrate the utility of imaging equipment and will potentially highlight a lack in quality or availability.
